# Supplementary material for: Attractive serial dependence arises during decision-making
Source: PLoS Biol. 2025 Aug 22;23(8):e3003333. doi: 10.1371/journal.pbio.3003333 (PMC12393773; doi:10.1371/journal.pbio.3003333)
Supplement: S1 Text — (DOCX) [file pbio.3003333.s001.docx]

**Supporting information**

To address the concern that the previous-target-induced attractive bias was actually driven by the rotated probe shown on the screen rather than driven by a higher-order decision-making process, we also conducted multivariate decoding of participants’ reports (that is, to decode the orientation of the report participants actually made instead of the target orientation that they should recall). If the attractive bias was driven by the rotated probe on screen, we should be able to see concurrent decoding of the participant’s report and the attractive neural bias, and the two should have the same posterior-centered topography because they were both stimulus-evoked. Decoding the participant’s report, we found four clusters with significant decoding after the probe onset. For these four time windows, only in one time window a marginally significant attractive bias was found (Fig S1; 1278 to 1814 ms after probe onset: t(19)=1.7479, p = 0.0966). For the other three (Fig S1; 214 to 822 ms, 2522 to 2766 ms after probe onset, and 1055 to 443 ms before participants completed recall), there was not a significant attractive bias (all *p*s > 0.4721).

For the 1278 to 1814 ms time window, searchlight analysis showed the previous-target-induced attractive bias is most prominent in right central sensors (Fig S1C), whereas the concurrent decoding of the participant’s report is most prominent in posterior sensors (Fig S1D). The searchlight topographies were nevertheless positively correlated (similarity between the two topographies measured with Pearson correlation, rho = 0.3978, *p* < 0.0001). While we therefore cannot rule out a contribution of the response to the trend towards a bias in this relatively late phase of the trial, it does not impact our main results, which focused on different time windows that were unaffected by the response.

**Replication in an EEG study**

To replicate the encoding-period repulsive bias reported in the previous study [1], in the EEG dataset we also investigated neural biases during encoding. Starting with the neural representation of sample 1, we focused on the decoding of sample 1 after its onset and before the onset of sample 2. For the previous-target-induced bias, we sorted trials according to the orientation of the previous target relative to sample 1. As shown in Fig S2A, we found no bias induced by the previous target: The cluster-based permutation test did not find any significant difference between asymmetry indexes. We further estimated the average bias during encoding (157-750 ms, from the start of significant decoding of sample 1 to the onset time of sample 2), and no significant bias was found (t(29) = -0.2921, p = 0.7723). For the neural representation of sample 2, we focused on the decoding of sample 2 after its onset and before the onset of the retrocue, and trials were sorted according to the previous target orientation relative to sample 2. Again, no significant bias was found either by the permutation test or averaging over the time window (Fig 8B, 893-1500 ms, from the start of significant decoding to the onset of retrocue, t(29) = 0.1670, p = 0.8685). The lack of repulsive neural bias could be due to the specific format of the feedback shown at the end of the previous trial and the noise masks presented before current-trial sample 1 (see Methods for a detailed description of the EEG task), which may have weakened the sensory adaptation caused by the previous target.

We further looked into the potential neural bias caused by sample 1 on the encoding of sample 2. Trials were sorted by the orientation of sample 1 relative to sample 2. Sample 1 repulsively biased the neural representation of sample 2 during its encoding stage (Fig 8D, 1077 - 1390 ms, p = 0.0032, cluster-based permutation test).

In sum, while we found no evidence for a repulsive bias from the previous trial’s target during encoding, we partially replicated the encoding-stage repulsive neural bias reported in our previous study [1].

**References**

1. Hajonides JE, Ede Fv, Stokes MG, Nobre AC, Myers NE. Multiple and Dissociable Effects of Sensory History on Working-Memory Performance. Journal of Neuroscience. 2023;43(15):2730-40.
