# Supplementary figures and images for: Attractive serial dependence arises during decision-making
Source: PLoS Biol. 2025 Aug 22;23(8):e3003333. doi: 10.1371/journal.pbio.3003333 (PMC12393773; doi:10.1371/journal.pbio.3003333)

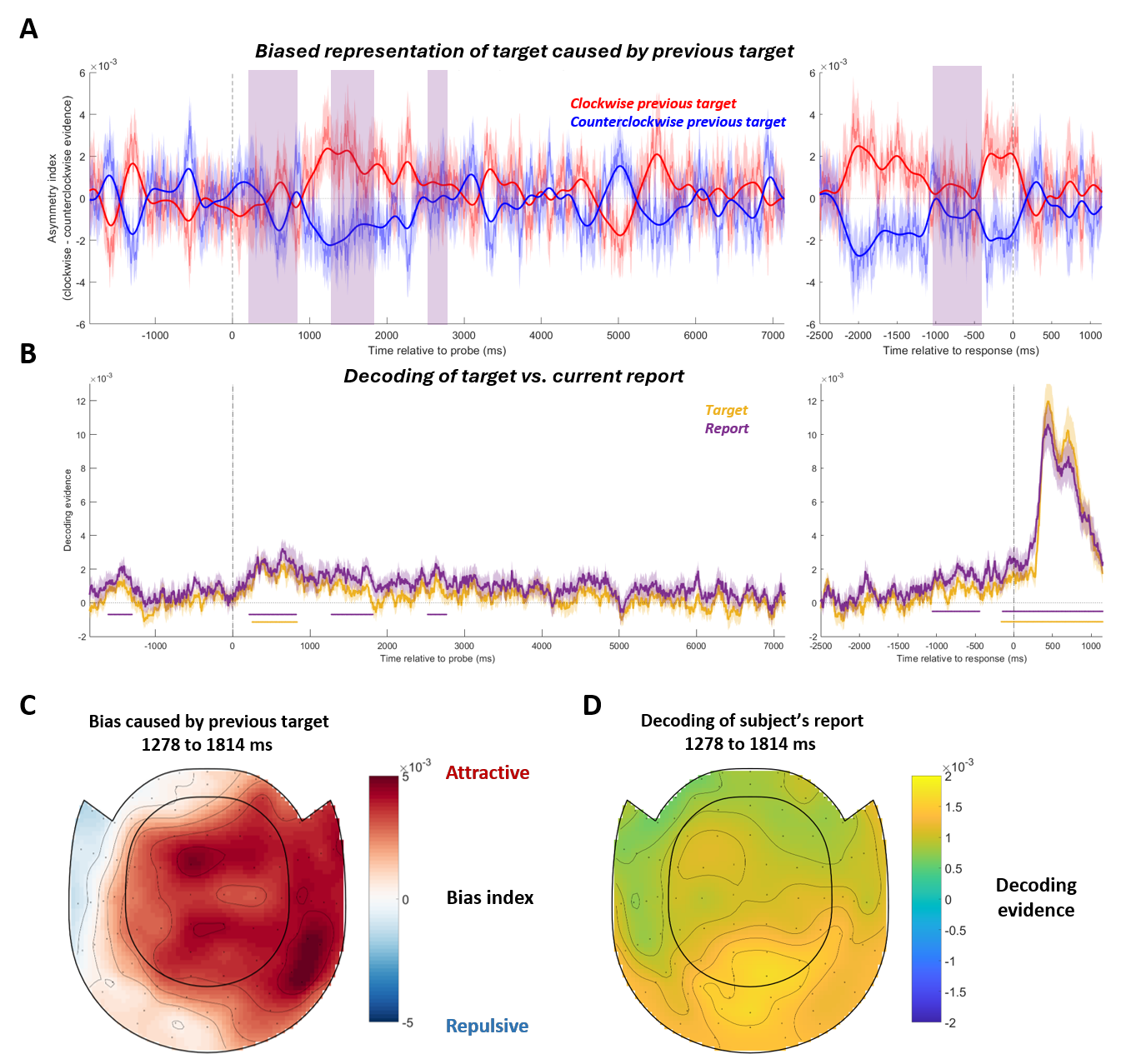

Supplement: S1 Fig — Yellow and purple horizontal lines in B indicate timepoints where a significant decoding of the target or the report orientation was found, respectively, as tested with cluster-based permutation tests. If the previous-target-induced attractive bias we found was merely driven by the rotated probe on the screen, we should be able to see a significant attractive bias in the time windows where the decoding of the report orientation is significant (purple-shaded regions in A). However, no significant attractive bias was found. In the only time window where a marginally significant previous-target-induced attractive bias was found, the topography of the attractive bias showed important contribution of the right central sensors (C) and the decoding of participant’s report is most prominent in posterior sensors (D). Plotting conventions are the same as in Fig 2. (TIF) [file pbio.3003333.s002.tif]

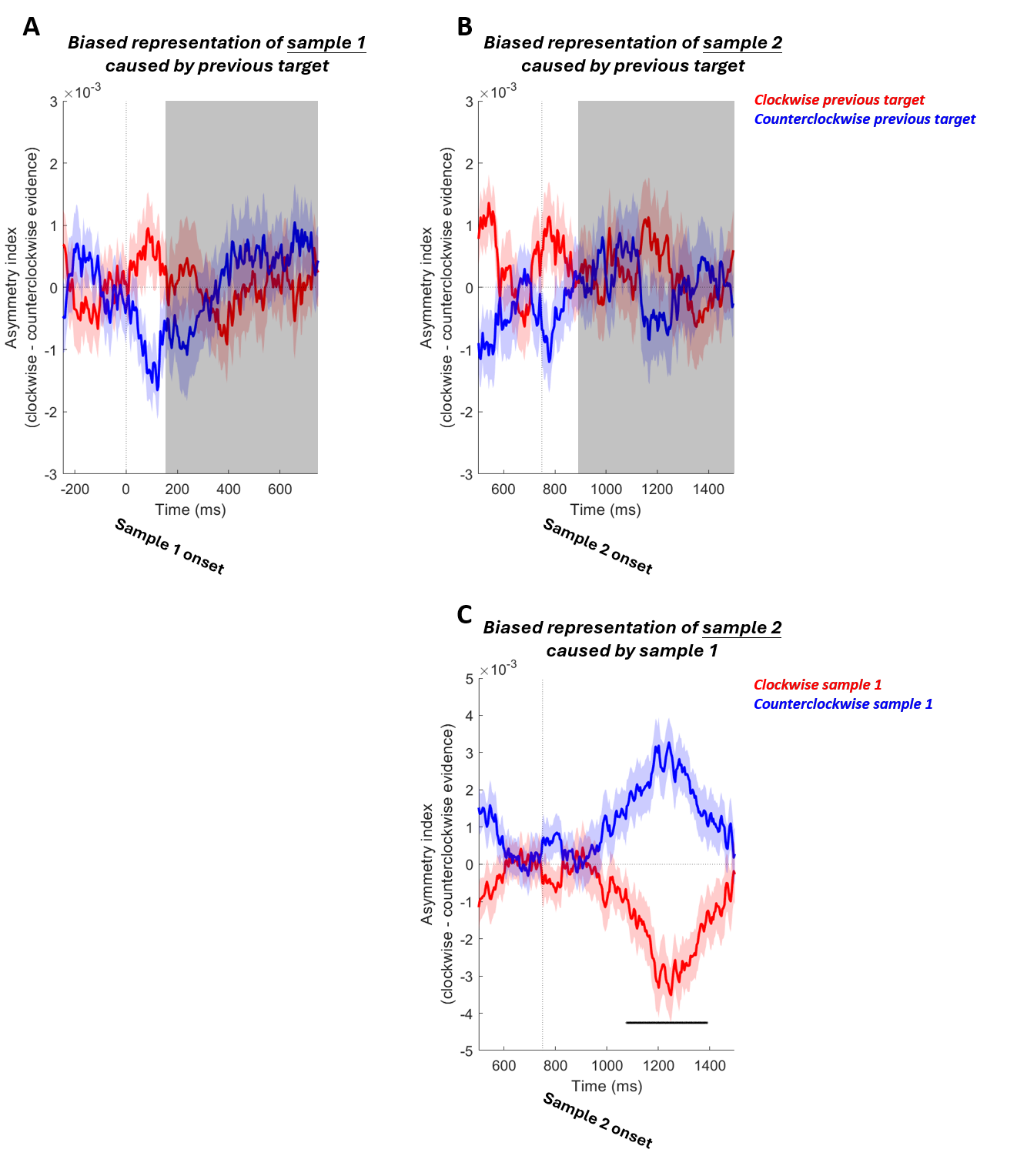

Supplement: S2 Fig — (A) Bias of the neural representation of sample 1 caused by the previous target. Trials were sorted according to whether the previous target was clockwise (red line) or counterclockwise (blue line) to sample 1. The y-axis shows the mean asymmetry index, with a positive number indicating a clockwise-biased neural representation. Shadings around the thin lines indicate SEM. The gray-shaded regions indicate time windows when sample decoding was significant and over which data were averaged for statistical inference. (B) Bias of the neural representation of sample 2 caused by the previous target. Trials were sorted according to the orientation of the previous target relative to sample 2. (C) Bias of the neural representation of sample 2 caused by sample 1. Trials were sorted according to the orientation of sample 1 relative to sample 2. The black horizontal line indicates timepoints with a significant bias assessed with cluster-based permutation testing. (TIF) [file pbio.3003333.s003.tif]
