# Supplementary material for: Attractive serial dependence arises during decision-making
Source: PLoS Biol. 2025 Aug 22;23(8):e3003333. doi: 10.1371/journal.pbio.3003333 (PMC12393773; doi:10.1371/journal.pbio.3003333)
Supplement: Attachment — Submitted filename: rv1_responses_to_reviewers.docx [file pbio.3003333.s006.docx]

REVIEWS:

Dear reviewers and editor,

We appreciate the thoughtful reviews and suggestions, which we believe have substantially improved our manuscript. We respond to individual points below. In particular we want to highlight that, per Reviewer 2’s recommendation, we identified a second dataset to replicate our main findings. We used a previously collected EEG dataset, in which participants did a working memory task with two memory items and a retrocue. We applied the same set of analyses we used on the MEG dataset on this EEG dataset. Since the dataset was not originally collected to examine serial biases the design was not optimized to replicate the effects we had already reported. Nevertheless, in this independent dataset, we replicated the key finding in the MEG study, namely that the previous target induced an attractive bias on the neural representation of the current target late in the trial but not during encoding. Interestingly, while the attractive neural bias again appeared late in the trial, it emerged earlier than in the MEG experiment, in the late maintenance delay. We discuss this difference below and in the manuscript, and believe it adds important nuance to our and other recently reported results (e.g., Luo et al., PLoS Biology 2025, Fischer et al., eLife 2024). Full details on results and methods are in the revised manuscript.

Reviewer #1: The authors present a new analysis of data showing serial dependence in representations of orientation during recall. They also find a repulsive effect for ignored stimuli. The experiment adds to growing evidence for serial dependence in memory processes. The experiment is creative, the results are interesting, and the manuscript is well written. I do have a number of major concerns, listed below, and these can be readily addressed with revised and moderated text. 
We very much appreciate the effort and careful thought that went into this review.

1.      The experiment design is more complicated than a traditional serial dependence experiment, which is fine, but it means some care is warranted in the interpretations. Unlike typical serial dependence (SD) experiments, stimuli are paired within single trials, along with added cues to selectively attend to one stimulus, and an attention task that encourages actively ignoring one sample in favor of the other. While some papers have used paired stimuli, and several serial dependence papers have studied the effect of attention, the design here is unique (in particular the added component of what some might consider active ignoring or suppressing). It's important to add caveats in the discussion that the complex pattern of results here may or may not extrapolate to all forms of serial dependence and may be a result of interactions between multiple mechanisms that involve different phenomena—more than just serial dependence.

Thanks for this suggestion. We’d like to note that the complicated design of this task affords an opportunity to study if and how different trial history (sample 1 and the previous target) influence the current recall differently, which we believe is a strength of this study.

We now discuss the possibility that participants might have used active suppressing or active removal of sample 1 (please see below our response to your Point #3).

2.      The feedback grating itself reinforces the target orientation. That could be an important source of a serial dependence signal. It's certainly an additional complication in the design that does not easily lend itself to simple interpretations. There is no response on that but it's highly correlated w target. Also highly correlated with response. And, no overt response is required for serial dependence to occur, as reported in many previous papers.

We agree that the feedback stimuli should enhance or reinforce the information of the target after the trial ended, hence potentially increasing its influence on the following trial.

We have now added in the Discussion (p.18) the possibility that the feedback may result in deeper processing of the previous target, possibly increasing serial dependence. It reads:

“Another important difference between the previous target and the sample 1 is that while the participants had recalled the previous target and received the feedback showing the correct orientation of the target after the recall, they did not need to recall sample 1. The recall and feedback may lead to deeper or prolonged processing of the previous target and putatively results in a greater impact on serial dependence. However, we believe it is unlikely that the attractive bias was driven purely by an action plan, since previous studies have shown that a response to the previous target is not necessary to produce serial dependence [4, 8, 9], and in the current tasks (both datasets) the action participants needed to conduct was independent of the actual orientation of the target (see Methods).”

3.      Several papers have investigated the role of attention in serial dependence. Most of these find that attention boosts serial dependence. The experiment here is related to those studies, but it requires more than just selective attention. In some conditions here, observers must actively suppress or ignore, such as when the second sample was cued, requiring observers to ignore or suppress the first sample. Interestingly, Rafiei et al., 2020, APP, found that actively ignored stimuli caused a *repulsive* bias. That is essentially what is found here in this experiment as well, and it could explain the finding that "when the second sample was cued it was repulsively biased away from the first sample on the same trial" (p. 3). The Rafiei paper seems especially relevant to the current manuscript.

Thanks for the suggestion! The possibility of active suppressing is quite interesting. Conceptually similar to active suppression, the behavioral results in our current study are also consistent with what was found in (Shan and Postle, 2022), where the information that was actively removed from working memory (WM) caused a repulsive serial bias. We have now added a more in-depth discussion of the possibility of actively suppressing (Rafiei et al., 2020) or removing (Shan and Postle, 2022) in the Discussion (p.18). It reads:

“The repulsive serial bias in behaviors has been reported in previous studies where a previously encoded item needs to be actively suppressed or actively removed. Rafiei and colleagues [39], for example, studied the serial dependence effect in an odd-one-out visual search task in which participants were required to find a target among distractors. While target orientation recall was attracted toward the target orientation in the previous trial, it was, at the same time, biased away from the mean orientation of the distractor ensemble. The orientation of distractors could be actively suppressed by the participants, and, as a result, cause a repulsive bias on the perception of the current target. Similarly, Shan and Postle [14] showed that while a no-longer-needed item that was removed from working memory by withdrawal of attention induced the classical attractive serial bias on the report in the following trial, an item that was actively removed instead induced a repulsive bias. In the current study, sample 1 became irrelevant when sample 2 was cued. Thus, participants could have used a strategy of active suppression or removal, causing the repulsive bias. However, at the mechanistic level, what happened in the current study may be different from active suppression/removal. Both active suppression and active removal are believed to exert their influence on a following stimulus during the perception/encoding of the new stimulus [39-41]. In contrast to this, in the current MEG experiment both sample 1 and the previous target caused repulsive neural biases when the new item was presented [1], and the difference between them emerged during decision-making, where the previous target, but not sample 1, induced an attractive neural bias. Similar to this, for the EEG experiment, the sample-1-induced repulsive bias happened when sample 2 was presented, which was before the participants knew which item to report, and thus before they knew which item they could suppress or remove from working memory. Hence, we believe the difference between the handling of sample 1 versus the previous target in both of our experiments happened during the recall but not the encoding stage.”

4.      More generally, the role of actively ignored things in serial dependence is under-studied and not well understood. This makes the manuscript more interesting, but it complicates interpretation and readers should be cautioned about this. The pattern of results could involve multiple mechanisms rather than the single encoding/decoding scheme proposed here. Because of the complicated design and possible involvement of multiple mechanisms, the conclusion that "attractive (but not repulsive) serial dependence arises during decision-making, and that priors that influence post- perceptual decision-making are updated by the previous trial's target, but not by other stimuli" may be an overgeneralized statement. It may only hold for this particular design.

We agree that it is possible that other mechanisms may be involved in our results. We have revised the manuscript to include the discussion about “active suppressing or removal” (see above our response to your Point #3).

We agree that this explanation does not generalize to all tasks on perception or working memory where serial dependence was found. However, a number of different designs (Fischer et al., 2024; Luo et al., 2025; Sheehan & Serences, 2022) have consistently found either repulsive effects at encoding, attractive effects during maintenance/recall, or both. While it is impossible to rule out that other designs will produce a different pattern, this points to at least some generalization in working memory tasks. Moreover, the inclusion of attention shifts via relevance or retro-cues and the presentation of distracting information may give these tasks greater relevance to how working memory is used in the real world.

We have now acknowledged the limited generalizability of our findings in the revised manuscript (p.16). It reads:

“While the current results speak for the two-stage models in which the attractive serial bias develops post-perceptually in a working memory task, they do not rule out the possibility that the attractive serial bias can emerge at the perceptual level, particularly in tasks focusing on perception rather than memory [34]. Many previous studies have found evidence showing that attractive serial dependence can occur at the time of perception [8, 22], or that it influences the early processing of visual information [21, 29]. Further studies are needed to understand when and how the attractive serial bias happens in a perceptual or post-perceptual stage.”

References

Fischer, C., Kaiser, J., & Bledowski, C. (2024). A direct neural signature of serial dependence in working memory. Elife. doi:10.7554/elife.99478.1

Luo M, Zhang H, Fang F, Luo H (2025) Reactivation of previous decisions repulsively biases sensory encoding but attractively biases decision-making. PLOS Biology 23(4): e3003150. <https://doi.org/10.1371/journal.pbio.3003150>

Sheehan, T. C., & Serences, J. T. (2022). Attractive serial dependence overcomes repulsive neuronal adaptation. PLoS biology, 20(9), e3001711. doi:10.1371/journal.pbio.3001711

5.      The authors should tone down claims in abstract and discussion about this adjudicating the debate between perception and memory. The results don't address that directly, and the design is a memory-centric one (involving lots of delays, retention intervals, multiple competing stimuli in working memory, feedback stimuli, etc). The results show a positive SD for recalled targets and a positive bias in activity. this could be due to attention, rehearsal (plus attention), or something else in memory, but it doesn't rule out that SD can happen in perception too. An experiment that generates a perception effect (an effect on appearance ) is necessary for that. The authors could either run additional experiments using other (simpler, less memory-focused) designs that would strengthen their claims, or they can tone down the overly broad speculation about perception vs memory.

Thanks for the suggestion! We want to clarify that we are not trying to adjudicate between effects in perception vs memory tasks, but whether serial effects in a memory task arise during encoding or later stages.

We have now acknowledged in the Discussion that the possibility of attractive SD arising from perception cannot be ruled out, and discussed the previous studies that support this view (please see above our response to your Point #4). We additionally clarify that our results pertain to serial dependence in memory rather than in perception.

6.      What about the other sample? It seems like repulsion only reported for sample 1. What about the fellow stimulus (the ignored one, report 1st, sample 2)? Was there repulsion of that? If not , the story isn't as simple as implied. Referring readers to the previously published paper for analyses of each condition isn't sufficient here, if the current manuscript is intended to be a complete and independent published paper. If this manuscript is just a follow-up analysis of one particular condition then maybe it is better as a letter to the editor at J Neurosci?

We argue that any bias induced by sample 2 on the report of sample 1 is retrospective and therefore should not be considered a serial dependence effect, as sample 1 was encoded before sample 2. We would instead interpret any influence of sample 2 on sample 1 as an inter-item bias between items concurrently held in WM. Thus, this is outside the scope of the current paper. At the neural level, sample 2 cannot influence the encoding of sample 1 via adaptation, so by definition any influence must occur through a different mechanism.

For completeness, we have now added the result of this sample-2-on-sample-1 bias analysis in the Results for the readers’ reference (p.3). It reads:

“For reference, we also examined the bias induced by sample 2 on the report of sample 1 in trials where both items were presented and sample 1 was cued. There was no significant sample-2-on-sample-1 bias (t(19) = 0.683, p = 0.5029). Please note, however, this bias should not be considered a serial dependence effect in the sense considered here as sample 1 was encoded before sample 2 and any influence would have been retrospective.”

7.      Results in Fig 4B and S1 cast doubt on the so-called response-based serial dependence, which has been reported in several papers, recently. Those papers claimed that serial dependence was the result of the report itself. The results here suggest otherwise. These results alone are very important to report, and should be highlighted in the discussion section as more than just a control, because they address an ongoing question in the literature.

Please note that the results reported in Fig 4B and S1 are showing the influence of the *current-trial* report orientation on the neural representation of the current-trial target. They are not investigating the possible bias induced by the report the subject made in the previous trial.

We chose not the study the influence caused by the previous report on the neural representation of the current target because the previous report is highly correlated with the previous target (as it should be). Thus, it is difficult to tell apart the contribution of previous report vs. previous target on the neural bias in the current trial. Any such analysis would require many trials to separately identify target- vs. response-related biases. Our study likely did not have sufficient statistical power for this subtle distinction.

8.      What are the decoding results for the feedback stimulus itself? Is it biased by the non-target inducer? Shouldn't it be, if the authors are correct about the repulsion effect?

The decoding of the feedback stimulus would be identical to the decoding of the current target (as shown in Fig. 2, starting from 200 ms after the report is made), because the feedback stimulus shows the correct orientation of the target.

Per your suggestion, we tested whether the neural representation of the feedback stimulus (i.e., the current trial target) was biased by sample 1. We used a time window of 200 to 1150 ms after the report (i.e., from feedback onset until the end of the epoch). We found no bias (t(19) = -0.7080, p = 0.4875). This could be due to the long time elapsed between sample 1 presentation and the presentation of the feedback, which is more than 3.6 sec plus the response time in each trial, in addition to the interference caused by sample 2 and by the response phase.

9.      In Fig 2A, the right graph shows decoding of target after response. Presumably this is decoding the feedback stimulus, no? If not, then it's unclear what "response" really means here. Is it not what the methods say? "Responses were submitted by pressing another button." This "another button" defines the response time? And it's what counts as "response," no? It's not clear what to take away from the right panel of 2A. But it seems possible that there could be a bias in this decoding as well.

Yes. It is the decoding of the feedback stimulus, as the feedback stimulus orientation is identical to the correct orientation of the target and appeared 200 ms after the response button press. We have now revised the legend of the figure 1 to clarify this.

The RT is determined by the time between the onset of the probe and when the subject pushed the “other button” to submit the report. The figure 1 legend, as well as the Methods, have now been revised to make this clearer to readers.

We analyzed the sample-1-induced bias on the neural representation of the feedback and found no bias there (see above our response to your Point #8).

10.     "We did not find any bias of the neural representation of the current target caused by the previous target (Fig 2B left; t = 0.4816, p = 0.6356). However, a bias seemed to arise later in the recall period (Fig 2B left, starting from ~1000 ms)." There does appear to be a clear effect at ~1000msec. Directly comparing the left and right panels of 2B is not exactly fair because the stimuli are very different in those plots. When the probe onset happens, it is a salient static stimulus. That there isn't a bias in decoding does not mean there is no bias in the representation—it's a null result, and it could simply be due to the low SNR of the biasing effect. That is, the positive sequential effect might manifest before t=1000, but it's not measurable because the probe stimulus has a lot of orientation energy that swamps the biasing signal. As the observer starts adjusting the probe, the orientations presented on the screen have increasing variability/noise until the point that
there is effectively a fully uniform distribution of orientations. In other words, moving the probe around in different ways on different trials adds noise and uncertainty that isn't present when the probe first appears. The authors sort of acknowledge this when they say that "the later part of the epoch contained increasing amounts of noise from irrelevant signals…" and they can test this themselves: plot stimulus orientations that the observers actually saw during the probe period. At time 0, there is no variance in orientation, because the random initial orientation of the probe is static at first. Once the observers start moving the probe around in different uncorrelated ways, there is increasing variability, though. That could add noise that actually makes the biased representation easier to detect. If the authors were to do another experiment where they just presented a white noise stimulus as the probe (rather than a high contrast, salient, noise free grating), they
might find that the previous target orientation could be decoded at t = 100 msec, not just t = 1000 msec. We can't know without another experiment, but the point is that trying to directly compare the response aligned data (Fig 2B right) versus probe onset data (Fig 2B left) isn't quite fair. And, absence of proof is not proof of absence, and claims about this being evidence for "decision" processes that take time are tenuous. Instead, a more direct experiment is called for.

Thanks for the question. We do not have the data of the orientation of the probe at every moment during the recall period. But we agree that the distribution of the probe orientation should become more uniform or random as the subject adjusted the probe to make the report, compared to the very beginning of the recall period when the probe had a static orientation.

We agree that the null effect of neural bias does not necessarily mean that there was no neural bias. It could be the case that the bias was there and we failed to detect it due to its low SNR. In the new EEG dataset (please see the very beginning of this Responses to Reviewers), where the decoding of the current target was overall stronger, we found clearer evidence for attractive neural bias during the late maintenance delay and recall.

We want to point out that we did not directly compare the results in the probe-locked analysis (Fig 2B left) and report-locked analysis (Fig 2B right). We do not argue that the attractive bias only happens very late in the decision-making. Instead, what is important in the time course is that the (presumably adaptation-related) repulsive neural bias was found in the time window where a significant decoding of current target was found. Thus, both the time course and the topography of the sample-1-induced neural bias (Fig 2A, 2C, 3A and 3B) suggested this repulsive bias is more likely to relate to the reactivation of the target (i.e., sample 2) and reflected the repulsive bias of the sample 2 neural representation which happened when it was encoded.

11.     Is accuracy related to the serial dependence effect? When observers were better at matching the target orientation, was there a stronger effect or more biased decoding? Is there a relationship between residual error (difference between reported orientation and feedback orientation) and sequential effect, biases measured or decoding bias?

Thanks for the question. Per your suggestion, we tested the relationship between the participant's performance in the task and the behavioral serial bias induced by sample 1 or previous target. For each participant, we calculated the mean absolute recall error in all trials (for the correlation with the previous-target-induced bias) and in both-shown-sample-2-cued trials (for the correlation with the sample-1-induced bias).

We observed a significant correlation between the absolute recall error and the previous-target-induced bias (Pearson’s r = 0.6465, p = 0.0021, see the left panel below), such that the stronger the attractive serial bias, the worse the recall performance. This suggests the more a participant relied on the previous target, the worse their performance is. For the sample-1-induced bias, we did not find a significant correlation (Pearson’s r = 0.3009, p = 0.1973, right panel).

Note: The correlation between mean absolute recall error and the serial-dependence induced by the previous target (left) or the sample 1 (right) across participants in the MEG dataset.

For the EEG dataset (see the very beginning of this Response to Reviewers), we tried the same analysis to see whether there was any relationship between the participant’s absolute recall error and the previous-target-induced behavioral bias. We did not assess the correlation with the sample-1-induced bias because there was no significant sample-1-induced bias in this dataset (please see the manuscript for details). We found that the relationship tended in the same direction as in the MEG dataset: a stronger attractive bias predicted higher absolute recall error (i.e., worse performance). This effect did not reach statistical significance (Pearson’s r = 0.2454, p = 0.1911; see below).

Note: The correlation between mean absolute recall error and the serial dependence induced by the previous target across participants in the EEG dataset.

These results have been reported in their respective Results section of the revised manuscript (p.3 and p.10).

12.     P. 4. "The target orientation could be decoded from 254 to 834 ms after probe onset" Is the feedback stimulus a problem for this analysis? What is RT? It's reported in methods, I think, but not in the main text. According to Fig 1, 250 msec after probe onset is the feedback stimulus in which "grating with the correct orientation was presented as feedback." Add some indication of RT in Fig 1, if that's the reason the feedback stimulus doesn't influence this decoding analysis. Also address it in the manuscript to avoid reader confusion.

The feedback stimulus should not be a problem for this analysis, because the feedback was always presented after the participants submitted their report (from 200 to 250 ms after the report was made), and 99% of the reports were made 700 to 6200 ms after probe onset (Methods p.20), meaning that feedback onset was no earlier than 900 ms after probe onset. The significant decoding of the target from 254 to 834 ms after probe onset is unlikely to be driven by this feedback.

The distribution of RTs is reported in the insert plot right below Fig 2A. To make it clearer to readers, we have now added the range of RT for 99% of trials in the figure legend as well.

13.     P. 12 "trails" should be "trials"

Thanks! We’ve corrected it in the revision.

14.     What happened to the single grating data (half the data according to Fig 1)? What was the behavioral effect for that?

Thanks for the question. It has been reported in the previous study (Hajonides et al., 2023) that the previous-target induced an attractive behavioral bias in all 4 groups of trials (only-first-item, only-second-item, two-item-report-first, and two-item-report-second trials).

In the current paper, for both the behavioral and neural effects, the single-grating trials were included in all previous-target-induced-bias analyses, along with the two-grating trials.

15.     The "reactivation" analyses are not entirely convincing because the stimuli on the screen are high contrast, noise-free, low uncertainty, etc, and could easily interfere or prevent measurement of reactivation even if it were present. Published papers reported that serial dependence is larger with more uncertain stimuli, in general, and so the reactivation may not be measurable not because it is absent but because it's washed out. Again, another example of arguing from null results.

Thank you for raising this possibility. We agree with you that the null effect of detecting a reactivation could be due to the strong signal from the salient probe that swamped the weaker signal of the previous target. We have now acknowledged this possibility in the Discussion. It reads:

“During the probe period we did not see any reactivation of the previous target in both datasets (Fig 5 and Fig 10), in contrast to [29, 30]. It could be that the weaker signal of the reactivated previous target was swamped by the stronger visual signal evoked by the probe that remained on the screen throughout the probe period, and, as a result, our decoding analyses failed to detect reactivation.”

16.     Not unrelated to the above, the stimuli in this experiment are not ideal for studying SD, if the goal is evaluating or making claims about SD in perception (as the discussion does). The gratings lack noise, apparently have high contrast, and have low uncertainty. Incidentally, the stimuli should be described in more detail: contrast, spatial frequency, size, etc.

Thanks for the suggestion! The stimuli used in the task are Gabor patches with diameter of 6° visual angle, two cycles per degree of visual angle, 50% contrast, and tapered by a Gaussian envelope with a 1.5° SD. We have now added a detailed description of stimuli in the Methods.

We have clarified that we do not claim that attractive serial bias cannot occur during perception, as is now acknowledged in the revised Discussion (see above our response to your Point #4). We believe it is possible that attractive bias can happen at different stages of the processing of information, and it is an important question for further studies to understand when and how attractive bias emerge in the perception/decision-making stage.

Relevant to your question here, we have also conducted the same set of analyses that we applied on MEG dataset to a new EEG dataset (please see the very beginning of this Responses to Reviewers). In that EEG dataset, the contrast of stimuli was the same as the MEG dataset (50%), but noise masks appeared in all delay periods. Similar to the results we got from the MEG experiment, in the EEG dataset, during the recall period we found an attractive neural bias induced by the previous target, but not the sample 1, on the neural representation of the current target. During the encoding of stimuli, we found a sample-1-induced repulsive neural bias on the representation of sample 2 when sample 2 was encoded. The full methods and results of this replication on the EEG dataset have been added to the revised manuscript. Please see it for details.

17.     P. 9. "An alternative proposal is that serial dependence of working memory representations develops post-perceptually" The "perception" versus "memory" origin of serial dependence is not a black-and-white alternative. It is possible that serial dependence operates at many levels and in many processes including working memory AND in perception and in other domains (like long term memory, affect, motor control, decision making, and across modalities). Kiyonaga et al., 2017 acknowledged this first but it's worth repeating here.

Thanks for the suggestion. We agree with you and have now emphasized this view in the Discussion (see above our response to your Point #4), in which we also cited (Kiyonaga et al., 2017).

18.     P. 9. "our finding suggests different previously attended information may not be incorporated into the prior to the same degree" This is also the point that the Rafiei et al APP 2020 paper made. Rather than arguing for encoding/decoding difference (p 10), what if the key difference in the experiment here is ignoring versus attending? The timecourse of ignoring (non-target) is different than the timecourse of attending  (target + feedback stimulus which reinforces the target) in this experiment, so it's not possible to use timecourse results or differences in timecourse to disentangle these alternatives.

Thanks for the question. We agree that the ignoring vs attending distinction is an interesting interpretation of our results. We discuss the possibility of ignoring/suppression in the revision (please see above our response to your Point #3). While we believe that ignoring vs attending could account for the distinct effect we saw during recall, we are reluctant to make a strong claim because we did not explicitly manipulate or instruct active removal or suppression of stimuli.

19.     Discussion section needs to be revised and the claims about perception vs memory toned down. The "perception" vs "post-perception" debate is not directly addressed by these experiments, in part because of the concerns above, and in part because the experiment design was geared specifically to tax memory processes (see comments above about complexity of the design, competing stimuli, retention intervals, rehearsal, feedback stimulus, etc). It is true that the experiment says something positive about memory processes. But, simply put, this experiment does not get at perception directly. A new experiment is needed with, for example, a detection task like that in Murai et al., Curr Bio, 2021 or Weilnhammer, et al., Curr Bio, 2023, or a forced choice experiment like Cicchini, et al, JoV, 2017, or one like Collins, JoV, 2020, or another simpler/more direct design that takes advantage of the fact that SD (in perception, though maybe not in memory) is retinotopically specific
(Collins, JoV, 2019). If new experiments are not added, then the discussion section should acknowledge these limitations and invite future work on the topic.

Thanks for the suggestion. We have revised the Discussion section to acknowledge that the results from the current study cannot rule out the possibility that attractive bias can arise via different mechanisms at the perceptual level, and we have cited studies supporting this view (see above our response to your Point #4).


Reviewer #2: The work here addresses a gap in knowledge about the time course and context of attractive and repulsive biases at the behavioral and neural level. Behavioral reports of orientation are often attracted towards the previously reported target. Moreover, in a visual working memory task with two oriented stimuli per trial (e.g., Hajonides et al. 2023 J Neuro), the orientation report of stimulus 2 is repulsively biased away from stimulus 1 and the previous target during encoding.

On its surface, a repulsive neural bias from the previous target during encoding seems incongruent with an attractive behavioral bias. But as the authors state, there is a lack of direct investigation of neural bias at the post-perceptual decision-making stage, where this discrepancy might be resolved. This is of theoretical interest because the popular 'continuity field' model predicts that attractive biases are the result of integrating the current stimulus with the prior stimulus early in perceptual processing. Thus, observing a late, post-perceptual locus of attractive bias would run counter to the dominant model in the field.

Here, the authors reanalyze data from a previous study (Hajonides et al. 2023 J Neuro), focusing on MEG data acquired during the memory-recall period (i.e., the decision-making stage) and make two key observations:

1.      The neural representation of the current target is attracted to the previous target during the decision-making stage — consistent with behavioral reports being attracted to the previous target.
2.      The neural representation of the current target is repelled from the other stimulus early in the decision making stage but is not attracted to the other stimulus from the current trial (i.e., there is only an attractive bias to the target on the previous trial). 

Thus, the authors find neural evidence for distinct early and late neural mechanisms for repulsive and attractive serial dependence during memory recall, respectively, and these observations are generally inconsistent with the continuity field model of serial dependence.

Many thanks for your careful reading of the manuscript and your questions and suggestions.

First, the authors find that the current target orientation can be decoded 254 to 834 ms after the probe's onset, and thus they treat this period as a time window of interest. However, this was not the temporal epoch where they observed an attractive bias: a bias arose later, ~1000 ms after the probe onset, much closer to the average response time (Fig 2A left). The target orientation was also significantly decodable 163 to 0 ms before the subjects indicated their final response (Fig 2A right), and an attractive bias was found during this narrow time window, including the last 2000 ms before a response was made.

Interestingly, during the early time window after the probe when the target was decodable, the authors observed a repulsive bias away from sample 1, consistent with the repulsion found in behavior. However, there was no bias induced by sample 1 in the response-locked analysis.

Introduction

"Moreover, in our previous study [1], different items in trial history induced different behavioral effects: Participants' report in the current trial was attracted toward the cued item from the previous trial (henceforth "previous target"), while the report of the second sample was repulsively biased away from the first sample in the same trial ("sample 1"). However, during encoding the neural representation of the current stimulus was repulsively biased away from both sample 1 and the previous target." (pg. 2)

Due to wording here, it's not clear whether sample 2 is both attracted towards the previous target and repulsed from the current sample 1, but it seems like Fig 1B indicates that it is?

Thanks for the question. Yes, the report of sample 2 is repulsed away from sample 1 and attracted toward the previous target at the same time. We have revised the wording here to make it clearer to readers. We did not break out the conditions to do the analysis for each pair of items, as it has been done and reported in (Hajonides et al. 2023). The attractive bias shown in Fig 1B is from all trials (both report-2 and report-1 trials). This is described in the Methods (p.20):

“For the sample-1-induced bias, we focused on trials where both items were presented and the second item was cued to be the target. For the previous-target-induced bias, we used all trials except the first trial in each block, as there was no previous target.”

Moreover, by this point in the manuscript, a reader might wonder whether sample 1 is biased by sample 2 in any way. Hajonides et al. 2023 J Neuro suggests no: "In trials with report first cues, there was no significant bias toward or away from the interfering second grating orientation that was not relevant to the task at hand (t(19)= 0.74, p = 0.467). In contrast, trials with report second cues revealed significant biases away from the initially encoded first grating orientation (t(19) = -2.33, p = 0.031; illustrated in Fig. 2B)."

We chose not to report this effect in the current paper because the influence caused by sample 2 on sample 1 is, by definition, not a serial dependence effect, as sample 1 was encoded before sample 2 was presented. If there was any sample-2-on-sample-1 bias, it would be an inter-item bias that happens between memory items in WM, instead of being a serial dependence effect. Hence, we don’t believe it is relevant to the topic of the current paper. (Please also see above our response to the Point #6 of Reviewer 1)

We have now added the result of this sample-2-on-sample-1 bias analysis in the Results for the readers’ reference (p.3). It reads:

“For reference, we also examined the bias induced by sample 2 on the report of sample 1 in trials where both items were presented and sample 1 was cued. There was no significant sample-2-on-sample-1 bias (t(19) = 0.683, p = 0.5029). Please note, however, this bias should not be considered a serial dependence effect in the sense considered here as sample 1 was encoded before sample 2 and any influence would have been retrospective.”

"Our goals were two-fold: First, we wanted to provide neural evidence that the attractive bias arises during the post-perceptual decision-making stage. Second, we wanted to show that this attractive bias is context-dependent — namely that only the target from the previous trial, but not the other stimulus from the current trial, led to an attractive neural bias." (pg. 2)

Shouldn't these goals be rephrased as hypotheses? This would really help frame the importance of the questions being asked, especially if put in the context of prominent models like the 'continuity field'. While the discussion does a good job of putting these findings in context, the intro is narrowly focused.

Thanks for the suggestion! We have now rephrased these goals as hypotheses in the Introduction (p.2):

“… However, during encoding the neural representation of the current stimulus was repulsively biased away from both sample 1 and the previous target. This neural effect at the encoding stage is contrary to the prediction of the continuity field theory of serial dependence, in which the attractive bias arises during perception or encoding into working memory of the new stimulus [4], but is consistent with the two-stage model [15]. Thus, we speculated that the discrepancy between the neural effect and behavioral report is resolved in the subsequent decision-making stage, as predicted by the two-stage model. In the current study, we re-analyzed the data from this study but focused on the memory recall period. We tested two hypotheses in our analyses: First, the neural evidence of attractive bias should arise during the post-perceptual decision-making stage. Second, this attractive bias should be context-dependent – namely that only the target from the previous trial, but not the other stimulus from the current trial, led to an attractive neural bias.”

Results

"Target decoding was significant from 163 to 0 ms before the response (Fig 2A right; p<0.00001)."

Why was neural evidence significant for such a long time after the response, relative to the duration of significance after the probe onset?

Resiliency during memory-recall? Or some mechanism that is preventing contamination of the target memory during reconstruction?

Thanks for the question. 200 ms after the participants made their responses (Fig. 1A), a feedback grating was shown on the screen for 50 ms, and it shows the correct orientation of the target in that trial. We believe the strong and significant decoding of the current target after the report was driven by this feedback stimulus.

We have now revised the legend of figure 2A to remind the readers about the feedback stimulus and the influence it had on the decoding of target.

"Fig 2. Decoding of the current target and the neural bias on the target induced by sample 1 or the previous target."

What biases would we expect to see if the trials were sorted by both the previous target and current sample 1? e.g., clockwise-previous-target-clockwise-sample-1 trials; clockwise-previous-target-counterclockwise-sample-1 trials. Do the biases interact?

Thanks for the question. We conducted a follow-up analysis to analyze the combined influence of sample 1 and the previous target on the neural representation of the current target. We focused on the trials where both samples were shown and sample 2 was cued to be the target. These trials were further broken out into four groups, by the relative orientation of the sample 1 and the previous target to the current target. So, for each participant and each group, we ended up with just ~20 trials, substantially reducing statistical power. In particular, this analysis only used ¼ of the trials used for the previous target analyses reported in the manuscript (i.e., 100 trials instead of 400).

The results from this analysis are therefore quite noisy (see the figures below).

Analysis locked to probe onset. Orange: clockwise previous target, clockwise sample 1. Purple: clockwise previous target, counter-clockwise sample 1. Yellow: counter-clockwise previous target, clockwise sample 1. Green: counter-clockwise previous target, counter-clockwise sample 1.

Analysis locked to report. Orange: clockwise previous target, clockwise sample 1. Purple: clockwise previous target, counter-clockwise sample 1. Yellow: counter-clockwise previous target, clockwise sample 1. Green: counter-clockwise previous target, counter-clockwise sample 1.

To increase the signal-to-noise ratio, we focused on the two time windows in the main manuscript (i.e., 254 to 834 ms after probe onset, and the 2000 ms before the report was made). We took the averaged asymmetry index in the time window and conducted a 2-by-2 repeated measures ANOVA, with the relative orientation (cw vs ccw) of the previous target and sample 1 as the factors.

For the time window shortly after the probe onset, we observed a main effect of the relative orientation of sample 1 (F(1,19)=19.423, p<0.001, see the left panel of the figure below), no main effect of the previous target (F(1,19)=0.294, p=0.594) and no interaction (F(1,19)=0.010, p=0.923).

For the time window before the participants submitted the report, we observed no main effect (sample 1: F(1,19)=1.295, p=0.269; previous target: F(1,19)=0.073, p=0.790, right panel) and no interaction (F(1,19)=0.559, p=0.464).

We also tried a similar analysis on the behavioral level: we again focused on all trials where both samples were shown and the sample 2 was cued. Trials were sorted into 4 groups according to the orientation of the sample 1 and the previous target relative to the target orientation (cw or ccw). The average recall error (calculated in a slightly way comparing to the analysis in the manuscript to make use of every trial we have: instead of calculating the serial bias with trials where the angle of the inducer item was [-45, 0] or [0, 45] deg relative to the target, we used all trials (i.e., two subgroups [-90, 0] or [0, 90] deg)) in each group was compared with a 2-by-2 repeated measure ANOVA. No significant main effect or interaction was found apart from a marginally significant effect of previous target (main effect of sample 1: F(1,19) = 0.432, p = 0.519; main effect of previous target: F(1,19) = 3.349, p = 0.083; interaction: F(1,19) = 0.567, p = 0.461; see figure below). Please note that this analysis also suffers from a lack of power, as only 100 trials per participants were taken into the analysis and they were further divided into four groups.

Methods

"Due to the higher level of noise in this data, none of the neural bias survived the cluster-based permutation test. Thus, we opted to testing the asymmetry indexes averaged in a time window with paired t-test."

In general, the effects are fairly subtle, and permutation testing is best practice. Do the authors have any other data sets (or can they find another open data set) that would partially replicate these findings? It is unlikely that there would be another paradigm with two stimuli on each trial, but there might be another study that at least has one target and a probe to assess probe-locked attractive biases?

Per your suggestion, we tried to replicate our findings and conducted the same set of analyses on a new dataset, which is from an EEG study with two memory items and a retrocue. With the new EEG dataset, we successfully replicated most of the key findings reported in the current study. To summarize the most important results, for the previous target, we found an attractive bias induced by it on the neural representation of the current target, and this happened in the decision-making stage (See the first figure below, panel A, as well as Figure 8 in the revised manuscript). For sample 1, we found a sample-1-induced repulsive bias on the neural representation of the sample 2 when sample 2 was encoded (panel C of the second figure below, as well as Fig S2 in the revised manuscript), but no repulsive bias during the decision-making stage (panel B of first figure below/Fig 8) or in behavior (Figure 6B in the revised manuscript). These findings replicated the effects in the MEG dataset, and align well the prediction of the two-stage model of serial dependence.

We have now reported the methods and results of this replication in the manuscript and discussed them in the Discussion. Please see the revised manuscript for details.

Note: This is the new figure 8 in the revised manuscript, showing the neural bias induced by the previous target (first row) and the sample 1 (second row) during the recall of target. (A) Bias of the neural representation of the current target caused by the previous target. Trials were sorted according to whether the previous target was clockwise (red line) or counterclockwise (blue line) to the current target. The y-axis shows the mean asymmetry index, with a positive number indicating a clockwise-biased neural representation. Shadings around the thin lines indicate SEM. The gray-shaded regions indicate time windows when sample decoding was significant and over which data were averaged for statistical inference. (B) Bias of the neural representation of the current target (i.e., sample 2) caused by sample 1 in sample-2-cued trials. Trials were sorted according to the orientation of sample 1 relative to the current target.

Note: This is the new figure S8 in the revised manuscript, showing the neural bias induced by the previous target (first row) and sample 1 (second row) during the encoding of the current stimulus. (A) Bias of the neural representation of sample 1 caused by the previous target. Trials were sorted according to whether the previous target was clockwise (red line) or counterclockwise (blue line) to sample 1. The y-axis shows the mean asymmetry index, with a positive number indicating a clockwise-biased neural representation. Shadings around the thin lines indicate SEM. The gray-shaded regions indicate time windows when sample decoding was significant and over which data were averaged for statistical inference. (B) Bias of the neural representation of sample 2 caused by the previous target. Trials were sorted according to the orientation of the previous target relative to sample 2. (C) Bias of the neural representation of sample 2 caused by sample 1. Trials were sorted according to the orientation of the sample 1 relative to the sample 2. The black horizontal line indicates timepoints with a significant bias assessed with cluster-based permutation testing.

"Principal component analysis was applied on this vector to reduce its dimensionality, maintaining 90% of the variance."

A few questions: how do you do PCA on a vector? How many components, on average, were retained? Does the choice of 90% matter for the results? And why use Mahalanobis distance on data after PCA given that the covariance matrix of the components is a diagonal matrix with 0s for off-diagonal elements? In this case, Mahalanobis distance reduces to the normalized Euclidean distance. I don't think this matters to the results, but it does make me wonder if I correctly understand the analysis pipeline.
Thanks for noticing this imprecise description. The PCA was applied on the matrix of data where each row of the matrix is the vector from one trial, i.e., the rows correspond to different observations (different trials in our experiment). Each column in the matrix corresponds to a different MEG sensor. We have now corrected this description in the manuscript (p.20).

Your understanding of our pipeline is correct. Applying the Mahalanobis distance after PCA is just getting the normalized Euclidean distance of the PCA scores.

The purpose of applying PCA and maintaining components accounting for 90% of the variance was to reduce the dimensionality of the raw data. With our MEG data, we have (306 sensors*37 time points in the sliding time window=) 11,322 dimensions. After reducing the dimensionality, around 160 features were retained and used to calculate the Mahalanobis distance. Skipping this dimensionality reduction step should not greatly influence the outcome of the analysis, as it has been done before with EEG (Muhle-Karbe et al. 2021; Wolff et al., 2017; Wolff et al., 2020; Zhang and Lewis-Peacock, 2024). We left it in because we have more sensors (306 compared to ~60 in EEG) and fewer trials, so to maintain a balance between the number of features and the number of observations we reduced the number of features via PCA.

For the second EEG dataset, in order to keep the analyses consistent with the MEG analyses, we chose to leave in the dimensionality reduction via PCA. As a results of that, around 31 features were retained and used to calculate the Mahalanobis distance.

We have now reported the number of components retained after PCA. You can find this information in the Methods (p.20). For the MEG dataset:

“To reduce its dimensionality, principal component analysis was applied to a matrix of data where each row corresponds to the vector from one trial, maintaining 90% of the variance. Across participants, 155.55±22.53 and 163.65±18.79 components were retained in the probe-locked and response-locked analyses, respectively.”

The number of components retained in the EEG dataset is also reported in the Methods (p.24):

“The decoding analyses were exactly the same as in the MEG dataset, with the exception that we did not exclude any trials due to a too-long or too-short RT. After PCA, across participants, 31.27±12.26 principal components were retained.”

**References**

Muhle-Karbe, P. S., Myers, N. E., & Stokes, M. G. (2021). A hierarchy of functional states in working memory. Journal of Neuroscience, 41(20), 4461-4475.

Wolff, M. J., Jochim, J., Akyürek, E. G., Buschman, T. J., & Stokes, M. G. (2020). Drifting codes within a stable coding scheme for working memory. PLoS biology, 18(3), e3000625. doi:10.1371/journal.pbio.3000625

Wolff, M. J., Jochim, J., Akyürek, E. G., & Stokes, M. G. (2017). Dynamic hidden states underlying working-memory-guided behavior. Nature neuroscience, 20(6), 864-871. doi:10.1038/nn.4546

Zhang, Z., & Lewis-Peacock, J. A. (2024). Reactivation of prior responses drives serial dependence and stabilizes working memory representations. The Journal of Neuroscience, e2399232024. doi:10.1523/JNEUROSCI.2399-23.2024

.
"To test whether the representation of the current target was biased. We looked into the
decoding results of the current target and took the mean Mahalanobis distances from channels
[-18°, -72°] and [18°, 72°] as clockwise and counterclockwise evidence, respectively. The
difference between them was calculated as the asymmetry index."

What motivates the choice of these bins, especially the inclusion of channels 72 degrees away? Based on the behavioral data in this study, and many others, I would expect that attractive serial dependence would peak around 30-40 degrees, so I didn't follow why these non-consecutive bins were used.

We apologize if we did not explain this clearly enough in the manuscript, but the bins used in the analysis are consecutive.

We constructed the neural tuning curve from 10 bins ([-90°, -72°], [-72°, -54°], [-54°, -36°], [-36°, -18°], [ -18°, 0°], [ 0°,18°], [18°, 36°],[36°, 54°],[54°, 72°], [72°, 90°]). In constructing the asymmetry index, for clockwise evidence we took the three channels [-72°, -54°], [-54°, -36°], [-36°, -18°], and for counterclockwise evidence we took another three channels, [18°, 36°],[36°, 54°],[54°, 72°]. This ensured sufficient sensitivity to detect a potential neural bias. Importantly, we expected that an inducer could shift the entire neural tuning curve, even if the inducer itself may have been most effective when it was ~40º from the target, which is why we averaged over all bins in the tuning curve that were unambiguously clockwise or counterclockwise relative to the target (i.e., excluding bins bordering 0º or 90º).

We have now modified this part in the Methods to make it clearer for the readers.

When we sorted trials into two groups according to the relative orientation of the inducer (ie. sample 1 or the previous target), we took in all trials which the inducer was [-90°, 0°] or [0°, 90°] relative to the current target. This was done to maximize the power of our analyses. We have also revised the Methods section regarding this part to make it clearer. It reads (p.21):

“Trials were separated into two groups according to whether the inducer orientation (i.e., previous target or sample 1) was clockwise relative to the current target (“clockwise inducer trials”, all trials where the inducer was [-90°, 0°] to the target) or counterclockwise relative to the current target (“counterclockwise inducer trials”, all trials where the inducer was [0°, 90°] to the target).”

Minor Comments

The font in the figures is very small, and gets fuzzy when magnified. Not a big deal here, but just something to remedy moving forward.

Thanks for highlighting this. The figures were compressed when the pictures were pasted into the word file (and potentially when the pdf was generated from the word). That is why the figures were so fuzzy. We have changed the setting of our word files to disable compression, and along with the revised manuscript, we have uploaded the .tiff files of the figures. They should be clearer and easier to read.

There were some minor typos:

"In the last 2000 ms before participants completed recall the previous target ..." (pg. 4)

"For the sample-1-induced bias, we focused on trial where …" (pg. 11)

"To analyzed the MEG activity in the probe period …" (pg. 11)

"We train the decoder on all non-rejected trials for the decoding of the current target." (pg. 11)

"Trails are sorted according to the orientation of the to-be-decoded item …" (pg. 12)

"To test whether the representation of the current target was biased. We looked into the decoding results of the current target …" (pg. 12)

"... the neural representation of current target is biased attractively toward the inducer, and vice versa." (pg. 12)

"... we use the same method but take the participant's report as the inducer." (pg. 12)

Thanks for noticing. We have now corrected all of them.


Reviewer 3:

Reviewer 3 noted that the paper is difficult to read and that important methodological details are missing, in particular when the authors refer to previous work without providing any details in the current manuscript.

We have now substantially revised the Methods and Introduction of the manuscript, per the suggestions raised by Reviewers 1 and 2, and added details previously reported in Hajonides et al., 2023. We hope this makes the paper easier to read.

In the Introduction:

- We now expand on the continuity field theory of serial dependence and how its predictions relate to the encoding stage results we previously reported (i.e., Hajonides et al., 2023) as well as how it predicts what should happen during the recall period (i.e., hypotheses of the current study).
- We rephrased the goal of the current study as hypotheses, to make them clearer. We more explicitly link our hypotheses to the predictions of two important models of serial dependence (the continuity field model and the two-stage model).

In the Methods:

- We have added detailed information about the stimuli used in the task (their contrast, radius, etc.)
- We have clarified how response times were calculated. In addition, in the legend of Figure 2, we also explicitly explained how the RTs relate to the time course of the neural decoding of the target.
- We clarified our description of how the PCA was calculated.
- We provide information about how many principal components were retained after PCA.
- We revised our description of the calculation of the asymmetry index, to enhance its clarity.
- We more explicitly explain how to interpret the asymmetry index with regard to the attractive or repulsive bias.

Furthermore, the indirect analyses and transformations on MEG data sometimes makes it challenging to interpret the findings. Reviewer 3's most important concern was that the metric used to measure similarity lacks a baseline. If the aim is to see a MEG signature of the serial bias, the authors need to demonstrate that the metric they choose in the first place reflects the target (without a bias).

We respectfully disagree with Reviewer 3’s comment. In Fig. 2A, we showed that we can get a reliable decoding of the target orientation with the method we used, which demonstrated that our neural decoding approach can reveal information about the current target without a bias.

Multivariate decoding of neural activity is a mature method that has been used for many years across different research fields in cognitive neuroscience, not just serial dependence (Bae & Luck, 2019; Fischer, Kaiser, & Bledowski, 2024; Hajonides, van Ede, Stokes, Nobre, & Myers, 2023; Luo et al., 2025; Sheehan & Serences, 2022), but also other perception- and working-memory-related fields (just to list a few examples: Sprague et al., 2018; Sprague, Ester, & Serences, 2014; Wolff, Jochim, Akyürek, Buschman, & Stokes, 2020; Wolff, Jochim, Akyürek, & Stokes, 2017; Yu, Teng, & Postle, 2020). The validity of this method has been widely demonstrated.

References

Bae, G.-Y., & Luck, S. J. (2019). Reactivation of Previous Experiences in a Working Memory Task. 30(4), 587-595. doi:10.1177/0956797619830398

Fischer, C., Kaiser, J., & Bledowski, C. (2024). A direct neural signature of serial dependence in working memory. Elife. doi:10.7554/elife.99478.1

Hajonides, J. E., van Ede, F., Stokes, M. G., Nobre, A. C., & Myers, N. E. (2023). Multiple and Dissociable Effects of Sensory History on Working-Memory Performance. 43(15), 2730-2740. doi:10.1523/JNEUROSCI.1200-22.2023 %J The Journal of Neuroscience

Luo M, Zhang H, Fang F, Luo H (2025) Reactivation of previous decisions repulsively biases sensory encoding but attractively biases decision-making. PLOS Biology 23(4): e3003150. https://doi.org/10.1371/journal.pbio.3003150Sheehan, T. C., & Serences, J. T. (2022). Attractive serial dependence overcomes repulsive neuronal adaptation. PLoS biology, 20(9), e3001711. doi:10.1371/journal.pbio.3001711

Sheehan, T. C., & Serences, J. T. (2022). Attractive serial dependence overcomes repulsive neuronal adaptation. PLoS biology, 20(9), e3001711. doi:10.1371/journal.pbio.3001711

Sprague, T. C., Adam, K. C. S., Foster, J. J., Rahmati, M., Sutterer, D. W., & Vo, V. A. (2018). Inverted Encoding Models Assay Population-Level Stimulus Representations, Not Single-Unit Neural Tuning. Eneuro, 5(3), ENEURO.0098-0018.2018. doi:10.1523/ENEURO.0098-18.2018

Sprague, Thomas C., Ester, Edward F., & Serences, John T. (2014). Reconstructions of Information in Visual Spatial Working Memory Degrade with Memory Load. Current Biology, 24(18), 2174-2180. doi:https://doi.org/10.1016/j.cub.2014.07.066

Wolff, M. J., Jochim, J., Akyürek, E. G., Buschman, T. J., & Stokes, M. G. (2020). Drifting codes within a stable coding scheme for working memory. PLoS biology, 18(3), e3000625. doi:10.1371/journal.pbio.3000625

Wolff, M. J., Jochim, J., Akyürek, E. G., & Stokes, M. G. (2017). Dynamic hidden states underlying working-memory-guided behavior. Nature neuroscience, 20(6), 864-871. doi:10.1038/nn.4546

Yu, Q., Teng, C., & Postle, B. R. (2020). Different states of priority recruit different neural representations in visual working memory. PLoS biology, 18(6), e3000769. doi:10.1371/journal.pbio.3000769
