# Supplementary material for: Attractive serial dependence arises during decision-making
Source: PLoS Biol. 2025 Aug 22;23(8):e3003333. doi: 10.1371/journal.pbio.3003333 (PMC12393773; doi:10.1371/journal.pbio.3003333)
Supplement: Attachment — Submitted filename: rv2_responses_to_reviewers_final.docx [file pbio.3003333.s007.docx]

Dear reviewers and editor, thank you for the time and effort you put into the review.

* We would like to suggest a different title to improve its accessibility for our broad audience: "Attractive serial dependence arises during decision-making". If you have an alternative suggestion, please reach out to me via email, so we can discuss the title before you re-submit your manuscript.

We have changed the title of this manuscript to "Attractive serial dependence arises during decision-making”. Thank you for the suggestion!

* Please add the links to the funding agencies in the Financial Disclosure statement in the manuscript details.

Done.

* Please include the approval/license number of the ethical approval for the experiments.

We have added ethics numbers for both experiments to the Methods.

* Please include information in the Methods section whether the study has been conducted according to the principles expressed in the Declaration of Helsinki.

We have now added the required information in the revision.

* DATA POLICY and CODE POLICY

We have uploaded the data and code for the current manuscript to the OSF repository (<https://osf.io/xd7rh>). The link to this repository is also available in the manuscript under the section “Data availability”.

Reviewer remarks:

Reviewer #1:
The authors did a great job revising this paper. It is much stronger and more interesting in many ways. There's new data, new analysis, a more nuanced discussion section, among other improvements. I thank the authors for their effort.

I still have some concerns, but I think these can be considered "minor". These can be fixed with modifications to the text, mostly in the discussion section. With these changes to the text, I think the manuscript will make a great addition to the literature.

Fig 3 shows attractive effect pretty clearly in posterior regions. Aside from a control analysis that isn't related, the fact remains that the attractive effect seems stronger in posterior regions, no?. Even if not during encoding, the effect is in sensory areas? How do the authors interpret this? It's not as clear a rejection or confirmation of any particular hypothesis. Are the  authors saying that there is attractive effect in decision making stage, which happens in posterior cortex?
Thank you for the question! We do believe the attractive neural bias shown in Fig 3 is prominent in the posterior brain, particularly for the repulsive bias shown in Fig 3a. While the topography for the attractive bias in Fig. 3c appears to be more distributed, we hesitate to draw strong conclusions in either direction from this pattern. At first blush, our posterior effect seems to be at odds with the frontal effect reported by Luo et al., 2025. However, our measure of bias (unlike theirs) is contingent on the presence of orientation decoding in the first place. Numerous studies have highlighted that visual memoranda are most strongly decodable in posterior regions (e.g., Myers et al., 2015, Wolff et al., 2020, consistent with the sensory recruitment hypothesis, see e.g., Postle, 2016). It is therefore difficult to say whether the posterior effect we observed is a mere result of decoding itself being most prominent in this area, even if the bias originates elsewhere. We can speculate that a biased representation is reinstated in posterior areas after the incorporation of biases elsewhere, but stress that our data cannot answer this question. We believe the reviewer has raised an interesting point that is worth highlighting as an avenue for future research.

We have now added clarification to the Results (page 7):

“… For the attractive bias found in the response-locked analysis, posterior sensors showed the largest bias (Fig 3C), again similar to the topography of target decoding in the same time window (Fig 3D; rho = 0.668, p < 0.0001), although right frontal areas also appeared to show an attractive bias. This suggests that the neural representation of the current target in posterior areas may be biased by the previous target when it is reinstated at the readout stage.”

We also touch on the point in the Discussion (page 17):

“One possibility is that the attractive bias gradually emerges throughout the delay period and decision-making stages. This could reconcile our results and reports of post-encoding attractive neural biases emerging at different task stages [30,31]. It might also explain why the attractive bias, once detectable in the MEG experiment, appeared largely at posterior sensors (Fig 3C), as it may have already incorporated the bias at this point and reinstated this biased representation in posterior areas. Whether this is indeed the case and, if so, sensory reinstatement is important for the development of the bias in behavior, is a question we cannot answer here.”

References

Myers, N. E., Rohenkohl, G., Wyart, V., Woolrich, M. W., Nobre, A. C., & Stokes, M. G. (2015). Testing sensory evidence against mnemonic templates. elife, 4, e09000.

Postle, B. R. (2016). Neural bases of the short-term retention of visual information. In P. Jolicoeur, C. Lefebvre, & J. Martinez-Trujillo (Eds.), Mechanisms of sensory working memory: Attention and performance XXV (pp. 43–58). Elsevier Academic Press.

Wolff, M. J., Jochim, J., Akyürek, E. G., Buschman, T. J., & Stokes, M. G. (2020). Drifting codes within a stable coding scheme for working memory. PLoS Biology, 18(3), e3000625.

P11. "While this contrasts with the significant across-trial repulsive neural bias
reported in in our previous study [1] it is still inconsistent with the emergence of the attractive bias during encoding."
This is concerning for a couple of reasons. First, it does not replicate the other study findings. Second, it is a null result: it could be there is an attractive bias that has nulled the repulsive one. If we believe the first study results, then only way to null those in these data would be with an additional attractive bias. Of course any number of other possibilities exist. Point is that it's a null result not a rejection of any hypothesis or "inconsistent" with anything in particular.

We have rephrased this claim to note merely the absence of positive evidence for an attractive bias at encoding and added a note in this part of the Results section (page 11) to direct the readers to the discussion:

“While this contrasts with the significant across-trial repulsive neural bias reported in in our previous study [1] (see Discussion for potential causes of the discrepancy), we still found no evidence for the emergence of the attractive bias during encoding.”

We have discussed the discrepancies between the results we got from experiment 2 and experiment 1 (both the results in the current manuscript and those reported in the previous study) and how they might be related to the differences in the different task designs between the two experiments. Specially for this result, please see page 17 in the Discussion section:

“The first stage of the two-stage model of serial dependence predicts a repulsive neural bias happening during the encoding of new stimuli. This effect has been shown in [1, 24, 30] but was not fully replicated in our EEG experiment: During the encoding of sample 2, we found that sample 1 repulsively biased the neural representation of this new sample. In contrast to the MEG dataset [1], however, we did not see a repulsive bias induced by the previous target during the current-trial encoding of either sample 1 or sample 2. This discrepancy could be explained by the specific format of the end-of-trial feedback showing the correct orientation of the target (which provided much weaker visual input in the EEG task compared to the MEG task), and the use of forward visual masks in the EEG study (which likely reduced the influence of the sensory adaptation to the previous target; see Methods). Despite the differences between the two experiments, neither experiment finds evidence for an attractive bias at encoding.”

P 12. "Compared to the attractive neural bias in the MEG experiment (which first emerged at ~ 1000 ms after probe onset, Fig 2B), the attractive bias in the EEG experiment started earlier in the trial (after retrocue onset, Fig 8A)." This is another departure and again merits more balanced discussion. The picture is not nearly as clear as the authors would like. A takeaway from the eeg data is that attractive effects could be present earlier but undetected in the meg experiment for a variety of reasons.

We agree that the attractive effects could emerge earlier in the delay at a point when decoding was too weak to be detected or was swamped by other stronger signals in the MEG experiment. This has been acknowledged in page 17 of the Discussion:

“Interestingly, the time course of the previous-target-induced attractive neural bias was different across the two experiments. In the MEG dataset, the attractive bias first emerged at ~1000 ms after the onset of the probe. In the EEG dataset, the attractive bias appeared to emerge after the retrocue onset. There were a number of task differences that could have led to this discrepancy. First, the target orientation was decodable in the late delay of the EEG experiment, possibly driven by increased signal from the presentation of noise stimuli. In the MEG experiment there was no target decoding in the delay. Since the attractive bias is contingent on the ability to decode the target, this may have led to the absence of a delay-period bias.”

Following the reviewer’s suggestion, we have expanded on this section, speculating that the bias may emerge (possibly gradually) throughout the delay (page 17):

“One possibility is that the attractive bias gradually emerges throughout the delay period and decision-making stages. This could reconcile our results and reports of post-encoding attractive neural biases emerging at different task stages [30,31].”

P14." Different from the results from the MEG analysis, in the EEG dataset we observed an attractive bias in the recall period, with a significant difference detected by the cluster-based permutation test (Fig 9, 4108 to 4357 ms, p = 0.0409)."
Again, an important difference meriting nuanced discussion and not big broad claims about serial dependence only happening in one stage or another or only at one time point or another. The results are very interesting and informative, but they are just not that decisive.

We chose not to belabor this difference because it was not central to our main hypotheses, as this analysis measured the neural bias of the participant’s upcoming report at the end of the current trial, not their previous report. We ran this analysis in both experiments to rule out that the previous-target-induced attractive neural bias could be caused by visual responses to the rotated probe during recall. This concern has been addressed by the analyses and results reported in the next section (Results section, page 14). There are a number of differences between the EEG and MEG experiments’ recall phases that could have caused this without affecting our main results – for example, the speed of probe rotation may have been different. Since we cannot be sure what caused the difference and it is not essential to our main aims, we hesitate to speculate. However, we would be happy to highlight the point if the reviewer thinks it would help make the manuscript clearer.

P 14 "but the attractive bias in the much shorter probe-onset-to-rotation-start
time window (see Fig 7A inset) failed to reach significance (t(29) = 1.6094, p = 0.1184"
This is clearly a decent effect size, it is positive, and it maybe even "moderate". Cohen's d nearly ~0.6? It doesn't reach the magic 0.05 p value level but it's a shorter time window, probably less statistical power, and perhaps the study is underpowered to actually address this question. It's clearly not a rejection, it's a positive trending result. An effect size that is "moderate" can't be ignored. It suggests there may be an attractive bias on the stimulus when it first appears, and it begs more experiments, more data. How is that effect size consistent with the late decision making story?
To be clear, having an effect this early would not disprove a later effect, but the later effect does not disprove the possibility of an earlier one either.

We agree that there may be a moderate attractive neural bias in this probe-onset-to-rotation-start time window. We believe this is consistent with our conclusion that the attractive bias was visible earlier in the EEG experiment, where decoding was generally stronger.

To acknowledge that the effect trends in the same direction as the effect measured from retrocue onset, we have rephrased the results section (page 14):

“In the much shorter probe-onset-to-rotation-start time window (see Fig 7A inset), the attractive bias trended in the same direction but failed to reach significance (t(29) = 1.6094, p = 0.1184).”

In light of the above, add some discussion material  about the mixed results above and trending effects and how these might impact the the debates in the literature. For example, one place to acknowledge the results above might be in the discussion section paragraph starting with "While the current results speak for the two-stage models in which the attractive serial bias develops post-perceptually in a working memory task, they do not rule out the possibility that the attractive serial bias can emerge at the perceptual level".

Thank you for the suggestion. In addition to the discussion about the differences between the two experiments that we already have in the manuscript, we have also addressed the potential origins of the differences between the MEG and EEG experiments in the next section (page 17):

“One possibility is that the attractive bias gradually emerges throughout the delay period and decision-making stages. This could reconcile the various differences in the results between our two experiments and reports of post-encoding attractive neural biases emerging at different task stages [30,31]. It might also explain why the attractive bias, once detectable in the MEG experiment, appeared largely at posterior sensors (Fig 3C), as it may have already incorporated the bias at this point and reinstated this biased representation in posterior areas. Whether this is indeed the case and, if so, sensory reinstatement is important for the development of the bias in behavior, is a question we cannot answer here.”

P 15 "This rules out that the observed attractive neural bias was driven by a reactivation of the previous target during decision-making." I think some readers might have concerns with the strength of this conclusion, because the reactivation signal could simply be too small to detect, or swamped. I think the authors acknowledged this well in the discussion section but the strong claim is made in the result section. So, the result section needs the acknowledgment too.

Thank you. We have modified this part of the results section to tune down the claim and direct readers to the Discussion section (page 15): “… This suggests that the observed attractive neural bias was unlikely to be driven by a reactivation of the previous target during decision-making (Please see Discussion).”

 Discussion. Sets up hypothesis A (one stage) vs hypothesis B (two stage). It's simple but the results aren't. Consider adding a third hypothesis C: both. The results are simply mixed (search for the word "discrepancy") and the two hypotheses are not actually mutually exclusive. It could be that repulsive and attractive effects happen at each stage? Or some other hybrid? Can the authors take this opportunity to speculate a bit about next-level accounts, future hypotheses, rather than trying to shoehorn clear differences in results into conforming to hypothesis B?

Thanks for the suggestion. We have now modified the Discussion to show this possible hybrid hypothesis (page 16):

“… For example, a recent model posits that serial bias is the result of a two-stage process: During the encoding of the new stimulus, trial history repulsively biases the encoded representation via sensory adaptation, and during readout of the memory representation, the trial history causes an attractive bias in a Bayesian-inference-like manner [7, 13, 15, 20]. These two hypotheses are not mutually exclusive, as a multiplicity of mechanisms may be creating both attractive and repulsive biases at several processing stages.”

Discussion " however, we did not see a
bias induced by the previous target during the current-trial encoding of either sample 1 or sample 2. This discrepancy could be explained by…" …Lots of things. One possibility is that attractive and repulsive effects are both present early but often dominated by repulsive ones simply bc of adaptation strength. That doesn't rule out the possibility of an attractive effect hiding in there and perhaps the discrepancy in the two studies is the sensitivity to the attractive effect.

Thank you. While the null bias in the EEG dataset did not replicate the repulsive bias found in the MEG dataset, we believe what is important here is that neither experiment finds evidence for an attractive bias during encoding, despite all the differences between the two experiments. We have added this clarification to the Discussion (page. 17):

“The first stage of the two-stage model of serial dependence predicts a repulsive neural bias happening during the encoding of new stimuli. This effect has been shown in [1, 24, 30] but was not fully replicated in our EEG experiment: During the encoding of sample 2, we found that sample 1 repulsively biased the neural representation of this new sample. In contrast to the MEG dataset [1], however, we did not see a repulsive bias induced by the previous target during the current-trial encoding of either sample 1 or sample 2. This discrepancy could be explained by the specific format of the end-of-trial feedback showing the correct orientation of the target (which provided much weaker visual input in the EEG task compared to the MEG task), and the use of forward visual masks in the EEG study (which likely reduced the influence of the sensory adaptation to the previous target; see Methods). Despite the differences between the two experiments, neither experiment finds evidence for an attractive bias at encoding.”

Title: in light of the results I think the title should change the word "underlying" to "contributions"
Thank you for the suggestion. Following the editor’s recommendation we have changed the title of this manuscript to "Attractive serial dependence arises during decision-making”.

Reviewer #2: Great job with the revision, and the inclusion of the new EEG data is a real strength.

Thank you!
